# Evidence for the Selective Reporting of Analyses and Discrepancies in Clinical Trials: A Systematic Review of Cohort Studies of Clinical Trials

**DOI:** 10.1371/journal.pmed.1001666

**Published:** 2014-06-24

**Authors:** Kerry Dwan, Douglas G. Altman, Mike Clarke, Carrol Gamble, Julian P. T. Higgins, Jonathan A. C. Sterne, Paula R. Williamson, Jamie J. Kirkham

**Affiliations:** 1 Department of Biostatistics, University of Liverpool, Liverpool, United Kingdom; 2 Centre for Statistics in Medicine, University of Oxford, Oxford, United Kingdom; 3 All-Ireland Hub for Trials Methodology Research, Queens University Belfast, Belfast, United Kingdom; 4 School of Social and Community Medicine, University of Bristol, Bristol, United Kingdom; 5 Centre for Reviews and Dissemination, University of York, York, United Kingdom; Georgetown University Medical Center, United States of America

## Abstract

**Background:**

Most publications about selective reporting in clinical trials have focussed on outcomes. However, selective reporting of analyses for a given outcome may also affect the validity of findings. If analyses are selected on the basis of the results, reporting bias may occur. The aims of this study were to review and summarise the evidence from empirical cohort studies that assessed discrepant or selective reporting of analyses in randomised controlled trials (RCTs).

**Methods and Findings:**

A systematic review was conducted and included cohort studies that assessed any aspect of the reporting of analyses of RCTs by comparing different trial documents, e.g., protocol compared to trial report, or different sections within a trial publication. The Cochrane Methodology Register, Medline (Ovid), PsycInfo (Ovid), and PubMed were searched on 5 February 2014. Two authors independently selected studies, performed data extraction, and assessed the methodological quality of the eligible studies. Twenty-two studies (containing 3,140 RCTs) published between 2000 and 2013 were included. Twenty-two studies reported on discrepancies between information given in different sources. Discrepancies were found in statistical analyses (eight studies), composite outcomes (one study), the handling of missing data (three studies), unadjusted versus adjusted analyses (three studies), handling of continuous data (three studies), and subgroup analyses (12 studies). Discrepancy rates varied, ranging from 7% (3/42) to 88% (7/8) in statistical analyses, 46% (36/79) to 82% (23/28) in adjusted versus unadjusted analyses, and 61% (11/18) to 100% (25/25) in subgroup analyses. This review is limited in that none of the included studies investigated the evidence for bias resulting from selective reporting of analyses. It was not possible to combine studies to provide overall summary estimates, and so the results of studies are discussed narratively.

**Conclusions:**

Discrepancies in analyses between publications and other study documentation were common, but reasons for these discrepancies were not discussed in the trial reports. To ensure transparency, protocols and statistical analysis plans need to be published, and investigators should adhere to these or explain discrepancies.

*Please see later in the article for the Editors' Summary*

## Abstract 2

In a systematic review of cohort studies, Kerry Dwan and colleagues examine the evidence for selective reporting and discrepancies in analyses between journal publications and other documents for clinical trials.

*Please see later in the article for the Editors' Summary*

## Editors' Summary

**Background:**

In the past, clinicians relied on their own experience when choosing the best treatment for their patients. Nowadays, they turn to evidence-based medicine—the systematic review and appraisal of trials, studies that investigate the benefits and harms of medical treatments in patients. However, evidence-based medicine can guide clinicians only if all the results from clinical trials are published in an unbiased and timely manner. Unfortunately, the results of trials in which a new drug performs better than existing drugs are more likely to be published than those in which the new drug performs badly or has unwanted side effects (publication bias). Moreover, trial outcomes that support the use of a new treatment are more likely to be published than those that do not support its use (outcome reporting bias). Recent initiatives—such as making registration of clinical trials in a trial registry (for example, ClinicalTrials.gov) a prerequisite for publication in medical journals—aim to prevent these biases, which pose a threat to informed medical decision-making.

**Why Was This Study Done?:**

Selective reporting of analyses of outcomes may also affect the validity of clinical trial findings. Sometimes, for example, a trial publication will include a per protocol analysis (which considers only the outcomes of patients who received their assigned treatment) rather than a pre-planned intention-to-treat analysis (which considers the outcomes of all the patients regardless of whether they received their assigned treatment). If the decision to publish the per protocol analysis is based on the results of this analysis being more favorable than those of the intention-to-treat analysis (which more closely resembles “real” life), then “analysis reporting bias” has occurred. In this systematic review, the researchers investigate the selective reporting of analyses and discrepancies in randomized controlled trials (RCTs) by reviewing published studies that assessed selective reporting of analyses in groups (cohorts) of RCTs and discrepancies in analyses of RCTs between different sources (for example, between the protocol in a trial registry and the journal publication) or different sections of a source. A systematic review uses predefined criteria to identify all the research on a given topic.

**What Did the Researchers Do and Find?:**

The researchers identified 22 cohort studies (containing 3,140 RCTs) that were eligible for inclusion in their systematic review. All of these studies reported on discrepancies between the information provided by the RCTs in different places, but none investigated the evidence for analysis reporting bias. Several of the cohort studies reported, for example, that there were discrepancies in the statistical analyses included in the different documents associated with the RCTs included in their analysis. Other types of discrepancies reported by the cohort studies included discrepancies in the reporting of composite outcomes (an outcome in which multiple end points are combined) and in the reporting of subgroup analyses (investigations of outcomes in subgroups of patients that should be predefined in the trial protocol to avoid bias). Discrepancy rates varied among the RCTs according to the types of analyses and cohort studies considered. Thus, whereas in one cohort study discrepancies were present in the statistical test used for the analysis of the primary outcome in only 7% of the included studies, they were present in the subgroup analyses of all the included studies.

**What Do These Findings Mean?:**

These findings indicate that discrepancies in analyses between publications and other study documents such as protocols in trial registries are common. The reasons for these discrepancies in analyses were not discussed in trial reports but may be the result of reporting bias, errors, or legitimate departures from a pre-specified protocol. For example, a statistical analysis that is not specified in the trial protocol may sometimes appear in a publication because the journal requested its inclusion as a condition of publication. The researchers suggest that it may be impossible for systematic reviewers to distinguish between these possibilities simply by looking at the source documentation. Instead, they suggest, it may be necessary for reviewers to contact the trial authors. However, to make selective reporting of analyses more easily detectable, they suggest that protocols and analysis plans should be published and that investigators should be required to stick to these plans or explain any discrepancies when they publish their trial results. Together with other initiatives, this approach should help improve the quality of evidence-based medicine and, as a result, the treatment of patients.

**Additional Information:**

Please access these websites via the online version of this summary at http://dx.doi.org/10.1371/journal.pmed.1001666.

- Wikipedia has pages on evidence-based medicine, on systematic reviews, and on publication bias (note: Wikipedia is a free online encyclopedia that anyone can edit; available in several languages)
- ClinicalTrials.gov provides information about the US National Institutes of Health clinical trial registry, including background information about clinical trials
- The Cochrane Collaboration is a global independent network of health practitioners, researchers, patient advocates, and others that aims to promote evidence-informed health decision-making by producing high-quality, relevant, accessible systematic reviews and other synthesized research evidence; the Cochrane Handbook for Systematic Reviews of Interventions describes the preparation of systematic reviews in detail
- *PLOS Medicine* recently launched a Reporting Guidelines Collection, an open-access collection of reporting guidelines, commentary, and related research on guidelines from across PLOS journals that aims to help advance the efficiency, effectiveness, and equitability of the dissemination of biomedical information

## Introduction

Selective reporting in clinical trial reports has been described mainly with respect to the reporting of a subset of the originally recorded outcome variables in the final trial report. Selective outcome reporting can create outcome reporting bias, if reporting is driven by the statistical significance or direction of the estimated effect (e.g., outcomes where the results are not statistically significant are suppressed or reported only as *p*>0.05) [1]. A recent review showed that statistically significant outcomes were more likely to be fully reported than non-significant outcomes (range of odds ratios: 2.2 to 4.7). In 40% to 62% of studies at least one primary outcome was changed, introduced, or omitted between the protocol and the trial publication [2]. Another review reached similar conclusions and also found that studies with significant results tended to be published earlier than studies with non-significant results [3].

Other types of selective reporting may also affect the validity of reported findings from clinical trials. Discrepancies can occur in analyses, if data for a given outcome are analysed and reported differently from the trial protocol or statistical analysis plan. For example, a trial's publication may report a per protocol analysis rather than a pre-planned intention-to-treat analysis, or report on an unadjusted analysis rather than a pre-specified adjusted analysis. In the latter example, discrepancies in analyses may also occur if adjustment covariates are used that are different to those originally planned. If analyses are selected for inclusion in a trial report based on the results being more favourable than those obtained by following the analysis plan, then analysis reporting bias occurs in a similar way to outcome reporting bias.

Examples of the various ways in which selective reporting can occur in randomised controlled trials (RCTs) have previously been described [4]. Furthermore, a systematic review of cohorts of RCTs comparing protocols or trial registry entries with corresponding publications found that discrepancies in methodological details, outcomes, and analyses were common [5]. However, no study to our knowledge has yet systematically reviewed the empirical evidence for the selective reporting of analyses in clinical trials or examined discrepancies with documents apart from the protocol or trial registry entry.

This study aimed to fill this gap by reviewing and summarising the evidence from empirical cohort studies that have assessed (1) selective reporting of analyses in RCTs and (2) discrepancies in analyses of RCTs between different sources (i.e., grant proposal, protocol, trial registry entry, information submitted to regulatory authorities, and the publication of the trial's findings), or between sections within a publication.

## Methods

### Study Inclusion Criteria

We included research that compared different sources of information when assessing any aspect of the analysis of outcome data in RCTs.

Cohorts containing RCTs alone, or a mixture of RCTs and non-RCTs were eligible. For those cohorts where it was not possible to identify the study type (i.e., to determine whether any RCTs were included), we sought clarification from the authors of the cohort study. Studies were excluded where inclusion of RCTs could not be confirmed, or where only non-RCTs had been included.

### Search Strategy

The search was conducted without language restrictions. In May 2013, the Cochrane Methodology Register, Medline (Ovid), PsycInfo (Ovid), and PubMed were searched (Text S2). These searches were updated on 5 February 2014, except for the search of the Cochrane Methodology Register, which has been unchanged since July 2012. Cochrane Colloquium conference proceedings from 2011, 2012, and 2013 were hand-searched, noting that abstracts from previous Cochrane Colloquia had already been included in the Cochrane Methodology Register. A citation search of a key article [1] was also performed. The lead or contact authors of all identified studies and other experts in this area were asked to identify further studies.

Two authors independently applied the inclusion criteria to the studies identified. Any discrepancies between the authors were resolved through discussion, until consensus was reached.

### Data Extraction

One author extracted details of the characteristics and results of the empirical cohort studies. Information on the main objectives of each empirical study was also extracted, and the studies were separated according to whether they related to selective reporting or discrepancies between sources. Selective reporting of analyses was defined as when the reported analyses had been selected from multiple analyses of the same data for a given outcome. A discrepancy was defined as when information was absent in one source but reported in another, or when the information given in two sources was contradictory. If selective reporting bias was studied, the definition of “significance” used in each cohort was noted (i.e., direction of results or whether the study used a particular *p*-value [e.g., *p*<0.05] to determine significance).

Data extraction was checked by another author. No masking was used, and disagreements were resolved through discussion.

### Methodological Quality

In the absence of a recognised tool to evaluate the methodological quality of the empirical studies eligible for this review, we developed and used three criteria to assess methodological quality:

1. Independent data extraction by at least two people
**High quality:** data extraction completed independently by at least two people.
**Methodological concerns:** data extraction not completed independently by at least two people.
**Uncertain quality:** not stated.
2. Definition of positive and negative findings
**High quality:** clearly defined.
**Methodological concerns:** not clearly defined.
**Not applicable:** this was not included in the objectives of the study.
3. Risk of selective reporting bias in the empirical study
**High quality:** all comparisons and outcomes mentioned in the methods section of the empirical study report are fully reported in the results section of the publication.
**Methodological concerns:** not all comparisons and outcomes mentioned in the methods section of the empirical study report are fully reported in the results section of the publication.

Two authors independently assessed these items for all studies. An independent assessor (Matthew Page) was invited to assess one study [6] because the first author was directly involved in its design. Any discrepancies were resolved through a consensus discussion with a third reviewer not involved with the included studies.

### Data Analysis

This review provides a descriptive summary of the included empirical cohort studies. We refrained from any statistical combination of the results from the different cohorts because of the differences in their design.

## Results

### Search Results

The search strategy identified 600 records. After duplicates were removed, 446 records were screened, and 390 were excluded. Full texts were accessed for 56 articles.

**Figure 1:**
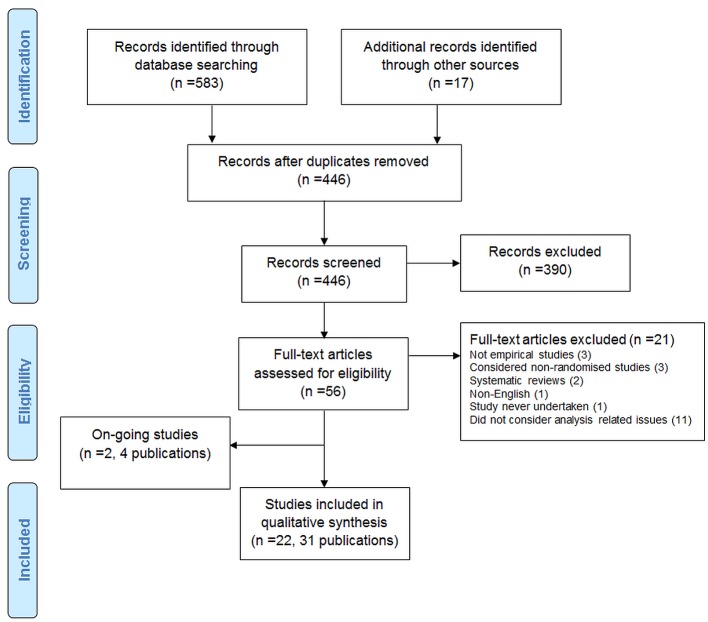
PRISMA flow diagram.

The PRISMA flow diagram is shown in Figure 1.

### Excluded Studies

Twenty-one articles were excluded after assessment of their full text: two were reviews; one was not in English; one was reported in abstract form only and the study was never undertaken; three were not empirical studies; three included only non-RCTs; and 11 did not consider analysis-related issues.

Two ongoing studies (four publications) were also identified [7]–[10]. One study [7],[9],[10] included 894 protocols (and 520 related journal publications) approved by six research ethics committees from 2000 to 2003 in Switzerland, Germany, and Canada. The aim of the study was to determine the agreement between planning of subgroups, interim analyses, and stopping rules and their reporting in subsequent publications. A conference abstract was identified in which the authors assessed RCTs submitted to the European Medicines Agency for marketing approval and assessed selective reporting of analyses [8]. The authors were contacted for further details.

### Included Studies

Twenty-two studies (in 31 publications) containing a total of 3,140 RCTs were included [6],[11]–[31].

All 22 included studies investigated discrepancies, and although several of these studies considered the statistical significance of results, none investigated selective reporting bias.

### Study Characteristics

Study characteristics are presented in Tables 1–3.

**Table 1:** Characteristics of included studies comparing protocols or trial registry entries to full publications.

Study	Objective	Cohort of Studies	Dates	Information Sources Compared	Included Study Designs and Number of Studies/Total Number of Studies	Funding Source for All Studies	Conclusions
**Al-Marzouki, 2008 [11]**	“To examine whether selective reporting of outcomes and subgroup analyses was present”	Trial protocols that had been peer reviewed and accepted for publication in *The Lancet*	Trial reports published by June 2007	Protocols to publications	71/75 RCTs; author obtained permission to use 64; 37 trials that had been published (50 reports) were assessed	Not stated	“The solution to the problem of selective reporting requires further discussion, the current system is clearly inadequate.”
**Chan, 2008 [15]**	“To evaluate how often sample size calculations and methods of statistical analysis are pre-specified or changed in randomised trials”	Protocols and journal publications of published randomised parallel group trials approved by the scientific ethics committees of Copenhagen and Frederiksberg, Denmark	Trials approved 1994–1995	Protocols to publications	70 RCTs	64% (45/70) industry, 16% (11/70) industry and non-industry, 14.3% (10/70) non-industry, 4.3% (3/70) none, 1.4% (1/70) unclear	“When reported in publications, sample size calculations and statistical methods were often explicitly discrepant with the protocol or not pre-specified. Such amendments were rarely acknowledged in the trial publication. The reliability of trial reports cannot be assessed without having access to the full protocols.”
**Hahn, 2002 [17]**	“To compare the outcomes, analysis and sample size proposed in the original approved study protocol with the results presented in the subsequent study report”	Approved protocols from a single local research ethics committee	Protocols approved in 1994	Protocols to publications	15/37 publications obtained; 2/15 RCTs	Not stated	“Our pilot study has shown that within-study selective reporting may be examined qualitatively by comparing the study report with the study protocol. Our results suggest that it [selective reporting] might well be substantial; however, the bias can only be broadly identified as protocols are not sufficiently precise.”
**Hernandez, 2005 [19]**	“To critically review the use of two types of secondary analyses, covariate adjustment and subgroup analysis, which are common in traumatic brain injury trials”	Published RCTs of TBI with >50 participants in each arm	Trial reports published 1966–2004	Protocols to publications for 6/18 RCTs; otherwise methods to results	18 RCTs	Not stated	“The reported covariate adjustment and subgroup analyses from TBI trials had several methodological shortcomings. Appropriate performance and reporting of covariate adjustment and subgroup analysis should be considerably improved in future TBI trials because interpretation of treatment benefits may be misleading otherwise.”
**Boonacker, 2011 [14]**	“To compare subgroup analyses as outlined in grant applications and their related publications”	Grants awarded by the Netherlands Organisation for Health Research and Development from 2001 onward that were finalised before March 1, 2010	Grants awarded 2001 to March 1, 2010	Grant proposals to publications and published protocols	47/79 RCTs, 13/79 cohort, 10/79 modelling, 9/79 other	100% Netherlands Organisation for Health Research and Development	“There is a large discrepancy between grant applications and final publications regarding subgroup analyses. Both non-reporting pre-specified subgroup analyses and reporting post-hoc subgroup analyses are common. More guidance is clearly needed.”
**Soares, 2004 [25]**	“To determine whether poor reporting of methods in randomised controlled trials reflects on poor methods”	RCTs conducted by the Radiation Therapy Oncology Group since its establishment in 1968	Terminated RCTs since 1968	Protocols to publication	59 RCTs	Not stated	“The reporting of methodological aspects of randomised controlled trials does not necessarily reflect the conduct of the trial. Reviewing research protocols and contacting trialists for more information may improve quality assessment.”
**Rosenthal, 2013 [6]**	“To evaluate discrepancies between trial registry entries and final reports of randomized controlled trials published in major general surgical journals”	RCTs published during 2010 in the *Annals of Surgery*, *Archives of Surgery*, and *British Journal of Surgery*	Trial reports published in 2010	Trial registry to publication	51 RCTs	37% (19/51) industry, 22% (11/51) no information, 41% (21/51) non-industry	“When interpreting the results of surgical RCTs, the possibility of selective reporting, and thus outcome reporting bias, has to be kept in mind. For future trials, prospective registration should be strictly respected with the ultimate goal to increase transparency and contribute to high-level evidence reports for optimal patient care in surgery.”
**Saquib, 2013 [24]**	“To assess adjustment practices for primary outcomes of randomized controlled trials and their impact on the results”	RCTs that reported primary outcome, published in print in 2009 in the 25 biomedical journals with the highest impact factor according to *Journal Citation Reports* 2009	Trial reports published in 2009	Trial registry, protocol, and design papers to publication	199 RCTs	Not stated	“There is large diversity on whether and how analyses of primary outcomes are adjusted in randomized controlled trials and these choices can sometimes change the nominal significance of the results. Registered protocols should explicitly specify adjustments plans for main outcomes and analysis should follow these plans.”
**Riveros, 2013 [31]**	“To compare the timing and completeness of results publicly posted at ClinicalTrials.gov and in published articles for trials of drug interventions”	RCTs of drugs with results posted on ClinicalTrials.gov and published	Results published on ClinicalTrials.gov by March 2012	Trial registry (results database) to publication	600 RCTs with results; 297 RCTs randomly sampled; 202 RCTs with posted and published results	85% (173/202) industry, 10% (20/202) academic, 5% (9/202) academic and industry	“The results highlight the need to assess trial results systematically from both ClinicalTrials.gov and the published article when available. Based on our results, searching Clinical Trials.gov is necessary for all published and unpublished trials to obtain more complete data and to identify inconsistencies or discrepancies between the publicly posted results and the publication.”

TBI, traumatic brain injury.

**Table 2:** Characteristics of included studies comparing information within a trial report.

Study	Objective	Cohort of Studies	Dates	Information Sources Compared	Included Study Designs and Number of Studies/Total Number of Studies	Funding Source for All Studies	Conclusions
**Cordoba, 2010 [16]**	“To study how composite outcomes, which have combined several components into a single measure, are defined, reported, and interpreted”	Parallel group RCTs published in 2008 reporting a binary composite outcome	Published in 2008	Abstract to methods to results	40 RCTs	40% (16/40) industry; 20% (8/40) partly industry, 18% (7/40) no industry, 23% (9/40) unclear	“The use of composite outcomes in trials is problematic. Components are often unreasonably combined, inconsistently defined, and inadequately reported. These problems will leave many readers confused, often with an exaggerated perception of how well interventions work.”
**Yu, 2010 [29]**	“To evaluate the use and reporting of adjusted analysis in randomised controlled trials (RCTs) and compare the quality of reporting before and after the revision of the CONSORT Statement in 2001”	Journal articles indexed in PubMed	Indexed on PubMed in December 2000 and December 2006	Methods to results	1,135 retrieved; 776 included; 197 reported adjusted analyses; 188 had a statistical methods section	Not stated	“The analyses specified in the methods section did not necessarily reflect how the results reported in the results section were obtained. Often the method was either not clearly specified or the results were obtained from different analyses from the specified ones.”
**Sun, 2011 [26]**	“The first is to describe the reporting of subgroup analyses and claim of subgroup effects. The second is to assess study characteristics associated with reporting of subgroup analyses, and with claims of subgroup effects. The third objective is to examine the analysis and interpretation of subgroup effects conducted for the primary outcome.”	Articles published in 118 core clinical journals (defined by the National Library of Medicine), randomly selected, stratified in a 1∶1 ratio by higher impact versus lower impact journals	Published in 2007	Methods to results	207/469 reported subgroup analyses	48% (99/207) industry, 52% (108/207) other	“Industry funded randomised controlled trials, in the absence of statistically significant primary outcomes, are more likely to report subgroup analyses than non-industry funded trials. Industry funded trials less frequently pre-specify subgroup hypotheses and less frequently test for interaction than non-industry funded trials. Subgroup analyses from industry funded trials with negative results for the primary outcome should be viewed with caution.”
**Wang, 2007 [30]**	“To assess the completeness and quality of subgroup analyses reported in *The New England Journal of Medicine*”	Original articles published in *The New England Journal of Medicine* that reported primary results from RCTs	Published July 1, 2005, through June30, 2006	Methods to results	97 RCTs in 95 articles; 59/97 reported subgroup analyses and were assessed further	Not stated	“When properly planned, reported, and interpreted, subgroup analyses can provide valuable information. With the availability of Web supplements, the opportunity exists to present more detailed information about the results of a trial. The purpose of the guidelines is to encourage more clear and complete reporting of subgroup analyses.”
**Hernandez, 2006 [18]**	“To review the appropriateness of reporting of subgroup analysis in RCTs recently published in major cardiology and internal medicine journals”	Phase 3 therapeutic cardiovascular RCTs with at least 100 patients, from journals with a high impact factor in the Institute for Scientific Information *Journal Citation Reports* 2002	Published September 1 to November 30, 2002, and May 1 to July 31, 2004	Methods to results	63 RCTs	Not stated	“Subgroup analyses in recent cardiovascular RCTs were reported with several shortcomings, including a lack of pre-specification and testing of a large number of subgroups without the use of the statistically appropriate test for interaction. Reporting of subgroup analysis needs to be substantially improved because emphasis on these secondary results may mislead treatment decisions.”
**Assmann, 2000 [12]**	“To assess the extent and quality of subgroup analyses in major clinical trial reports”	A sample of 50 consecutive clinical trial reports were obtained from four major medical journals (*British Medical Journal, Journal of the American Medical Assocation, The Lancet, The New England Journal of Medicine*) during July to September 1997	Published July to September 1997	Methods to results	50 RCTs	Not stated	“Clinical trials need a predefined statistical analysis plan for uses of baseline data, especially covariate-adjusted analyses and subgroup analyses. Investigators and journals need to adopt improved standards of statistical reporting, and exercise caution when drawing conclusions from subgroup findings.”
**Bhandari, 2006 [13]**	“To evaluate the current use of baseline comparability tests and subgroup analyses in surgical randomized controlled trials”	Published surgical RCTs in four medical journals (*British Medical Journal, Journal of the American Medical Assocation, The Lancet, The New England Journal of Medicine*) and in the *Journal of Bone and Joint Surgery* (American and British volumes)	Published January 2000 to April 2003	Methods to results	72 RCTs	Not funded 19.4% (14/72), not stated 1.4% (1/72), funded 79.2% (57/72) Funded by: not reported, 2; industry, 24; peer reviewed agency, 36; charity, 3; internal, 10	“We identified important problems with the reporting of randomization, baseline comparisons, and subgroup analyses. The most concerning was the misuse of subgroup analyses. One in three studies did a subgroup analysis, of which most found subgroup differences using inappropriate statistical tests. The presentation of these subgroup findings in the conclusions only exaggerates the perceived significance of such exploratory analyses.”
**Moreira, 2001 [21]**	“To show how often inaccurate or incomplete reports of subgroup analysis occur”	Published RCTs in four leading journals, i.e., *The New England Journalof Medicine, Journal of the American Medical Association, The Lancet*, and the *American Journal of Public Health*; eight consecutive reports from each journal published after July 1, 1998	Published after July 1, 1998	Methods to results	32 RCTs	Not stated	“Current reporting of subgroup analysis in RCT is incomplete and inaccurate. The results of such subgroup analysis may have harmful effects on treatment recommendations if accepted without judicious scrutiny.”
**Parker, 2000 [22]**	“To determine how subgroup analyses are performed in large randomized trials of cardiovascular pharmacotherapy”	Double-blind RCTs of pharmacotherapy with clinical outcomes as primary end points, published between 1980 and 1997; trials with <1,000 patients were excluded to ensure a sizeable number of patients in the selected subgroups	Published between 1980 and 1997	Methods to results	67 RCTs	Not stated	“Trial subgroups should ideally be defined a priori on two bases: single-factor subgroups with a strong rationale for biological response modification and multifactorial prognostic subgroups defined from baseline risks. However, single-factor subgroup analyses are often reported without a supporting rationale or formal statistical tests for interactions. We suggest that clinicians should interpret published subgroup-specific variations in treatment effects sceptically unless there is a pre-specified rationale and a significant treatment-subgroup interaction.”

**Table 3:** Characteristics of included studies comparing other documentation to full publications.

Study	Objective	Cohort of Studies	Dates	Information Sources Compared	Included Study Designs and Number of Studies/Total Number of Studies	Funding Source for All Studies	Conclusions
**Melander, 2003 [20]**	“To investigate the relative impact on publication bias caused by multiple publication, selective publication, and selective reporting (ITT versus PP) in studies sponsored by pharmaceutical companies”	Placebo-controlled studies of five selective serotonin reuptake inhibitors submitted to the Swedish drug regulatory authority as a basis for marketing approval for treating major depression	Submitted between 1989 and 1994	Study reports in marketing approval compared to publications	42 RCTs	100% industry	“The degree of multiple publication, selective publication, and selective reporting differed between products. Thus, any attempt to recommend a specific selective serotonin reuptake inhibitor from the publicly available data only is likely to be based on biased evidence.”
**Rising, 2008 [23]**	“To determine the publication rate of efficacy trials submitted to the Food and Drug Administration (FDA) in approved New Drug Applications (NDAs) and to compare the trial characteristics as reported by the FDA with those reported in publications”	All efficacy trials found in approved NDAs for new molecular entities and all published clinical trials corresponding to the trials within the NDAs	From 2001 to 2002	NDA to publication	164 efficacy trials found in 33 NDAs	77% (97/126) at least in part by industry	“Many trials were still not published 5 years after FDA approval. Discrepancies between the trial information reviewed by the FDA and information found in published trials tended to lead to more favourable presentations of the NDA drugs in the publications. Thus, the information that is readily available in the scientific literature to health care professionals is incomplete and potentially biased.”
**Turner, 2008 [27]**	“To determine how accurately the published literature conveys data on drug efficacy to the medical community by comparing drug efficacy inferred from the published literature with drug efficacy according to FDA reviews”	Phase 2 and 3 clinical trial programs for 12 antidepressant agents approved by the FDA	Approved by the FDA between 1987 and 2004	FDA review to publication	74 FDA-registered studies	100% industry	“We cannot determine whether the bias observed resulted from a failure to submit manuscripts on the part of authors and sponsors, from decisions by journal editors and reviewers not to publish, or both. Selective reporting of clinical trial results may have adverse consequences for researchers, study participants, healthcare professionals, and patients.”
**Vedula, 2013 [28]**	“To compare the description of types of analyses and criteria for including participants in the publication (i.e., what was reported) with descriptions in the corresponding internal company documents (i.e., what was planned and what was done)”	Trials of gabapentin funded by Pfizer and Warner-Lambert's subsidiary Parke-Davis for off-label indications	Published 1987–2008	Internal company documents (statistical analysis plan, protocol, and research report) to trial report	11/21 published studies	100% industry	“Descriptions of analyses conducted did not agree between internal company documents and what was publicly reported. Internal company documents provide extensive documentation of methods planned and used, and trial findings, and should be publicly accessible. Reporting standards for randomized controlled trials should recommend transparent descriptions and definitions of analyses performed and which study participants are excluded.”

FDA, US Food and Drug Administration; ITT, intention to treat; NDA, new drug application; PP, per protocol.

Included cohort studies were published between 2000 and 2013: two compared marketing approval or new drug applications to publications; one compared US Food and Drug Administration reviews to publications; one compared internal company documents to publications; five compared protocols to publications; two compared trial registry entries to publications; one compared grant proposals and protocols to publications; one compared trial registry entries/protocols/design papers to publications; and nine compared information within the trial report (i.e., between sections such as the abstract, methods, and results). Of the cohort studies, 91% (20/22) included only RCTs, with a median of 61 RCTs per cohort study (interquartile range: 41 to 91; range: 2 to 776).

Included RCTs were published between 1966 and 2012. The source of funding of RCTs was not considered in 11 of the cohort studies. In ten studies, industry funded a median of 70.5% of the RCTs (interquartile range: 46% to 100%). In one study, all of the RCTs were funded by the Netherlands Organisation for Health Research and Development.

### Methodological Quality

Details of the methodological quality of the included studies are presented in Table 4.

**Table 4:** Methodological quality.

Study	Independent Data Extraction by Two People		Positive and Negative Findings Clearly Defined		Selective Reporting	
**Melander, 2003 [20]**	Uncertain quality	Not stated	High quality	Statistically significant/non-significant	High quality	All results reported on stated objectives
**Al-Marzouki, 2008 [11]**	Uncertain quality	Not stated	NA	Not considered	High quality	All results reported on stated objectives
**Chan, 2008 [15]**	High quality	Two reviewers used electronic forms to independently extract data; resolved disagreements by discussion	NA	Not considered for sample size or statistical analysis although considered when looking at outcomes	High quality	All results reported on stated objectives
**Cordoba, 2010 [16]**	High quality	Two independent observers extracted the data using a standardised data sheet, and two other observers, blinded to the results, selected the most important component	NA	Not considered	High quality	All results reported on stated objectives
**Rising, 2008 [23]**	High quality	One author extracted all data, and additional coders were trained to double-code portions of the data determined to be potentially subjective in both the FDA reviews and the publications; disagreements were resolved by consensus	High quality	Favourable: statistically significant in favour of the drug; not favourable: not statistically significant or significant in favour of the comparator	High quality	All results reported on stated objectives
**Turner, 2008 [27]**	High quality	Double data extraction was used; any discrepancies were resolved by consensus	High quality	Positive: statistical superiority (*p*<0.05) to a comparator, usually placebo	High quality	All results reported on stated objectives
**Yu, 2010 [29]**	Methodological concerns	Two authors extracted trial characteristics, but only one extracted outcome data	High quality	Statistically significant/not significant	High quality	All results reported on stated objectives
**Sun, 2011 [26]**	High quality	Teams of two reviewers independently abstracted data using standardised pilot-tested extraction forms; discrepancies resolved by consensus	High quality	Statistically significant/not significant—assessed for primary outcome and for association with reporting of subgroup analyses	High quality	All results reported on stated objectives
**Vedula, 2013 [28]**	High quality	One author extracted data and another verified, with a third person verifying discordant items	High quality	*p*<0.05 indicates statistical significance	High quality	All results reported on stated objectives
**Hahn, 2002 [17]**	Methodological concerns	All three authors assessed three studies to check for consistency, and then the first author assessed the other studies	NA	NA	High quality	All results reported on stated objectives
**Wang, 2007 [30]**	Uncertain quality	Not stated	High quality	Significant: finding a nominally significant difference among treatment groups in a superiority trial or declaring non-inferiority/equivalence in a non-inferiority/equivalence trial	High quality	All results reported on stated objectives
**Hernandez, 2006 [18]**	High quality	Data extraction completed independently by two authors	High quality	Referred to as negative/positive; negative defined as non-significant overall result	Methodological concerns	Not all secondary analyses reported—only that a difference was observed (between positive and negative trials)
**Assmann, 2000 [12]**	High quality	Data extraction appears to have been completed independently by two authors	NA	NA	High quality	All results reported on stated objectives
**Bhandari, 2006 [13]**	High quality	Data extraction was completed independently by three authors	NA	NA	High quality	All results reported on stated objectives
**Hernandez, 2005 [19]**	Methodological concerns	Data extraction performed by one author	Methodological concerns	Positive and negative used but not defined	High quality	All results reported on stated objectives
**Moreira, 2001 [21]**	Methodological concerns	Data extraction performed by one author	NA	NA	High quality	All results reported on stated objectives
**Parker, 2000 [22]**	Uncertain quality	Not stated	High quality	A positive outcome means that the treatment was beneficial and the treatment effect achieved nominal statistical significance; a negative/equivocal outcome means that a significant benefit was not demonstrated	High quality	All results reported on stated objectives
**Boonacker, 2011 [14]**	Uncertain quality	Not stated	High quality	A subgroup analysis was defined as significant when the researchers reported a significant effect by either (1) providing a significant *p-*value for the interaction test and/or (2) reporting the results of the stratified analyses, whereby confidence intervals differed significantly between the subgroups, and/or (3) stating that there was a significant subgroup effect without providing the actual numerical values	High quality	All results reported on stated objectives
**Soares, 2004 [25]**	Uncertain quality	Not stated	NA	NA	High quality	All results reported on stated objectives
**Rosenthal, 2013 [6]**	Methodological concerns	An internal pilot study was conducted to test the data extraction spreadsheet for feasibility, completeness, and accuracy, and as a quality control check, data entries concerning these RCTs were cross-checked by the second reviewer	NA	NA	High quality	All results reported on stated objectives
**Saquib, 2013 [24]**	High quality	Two researchers independently extracted data and discussed discrepancies to reach a consensus, otherwise the senior investigator was consulted	High quality	Nominal statistical significance, based on 95% confidence intervals being entirely on one side of the null, *p*<0.05, or a statement in the text	High quality	All results reported on stated objectives
**Riveros, 2013 [31]**	High quality	All data were extracted in duplicate by two eviewers in data collection forms; all disagreements were resolved by discussion to reach a consensus, including intervention of a third reviewer in case of discrepancies	NA	NA	High quality	All results reported on stated objectives

FDA, US Food and Drug Administration; NA, not applicable.

#### Independent data extraction by at least two people

We had methodological concerns about five studies because data extraction was completed by only one person or only a sample was checked by a second author. Eleven studies were high quality, as data extraction was completed by at least two people. Six studies were rated as uncertain quality, as information regarding data extraction was not provided.

#### Definition of positive and negative findings

We had methodological concerns about one study because positive and negative findings were not defined, while 11 studies were of high quality, with clear definitions of positive and negative findings. Defining a positive or negative finding was not a study objective in ten studies.

#### Risk of selective reporting bias in the empirical study

One study [18] generated methodological concerns because some secondary analyses were not reported, and the study report stated only that no difference was observed between positive and negative trials. The remaining 21 studies were of high quality, with all comparisons and outcomes stated in the study methods reported in full. We did not have access to any protocols for the empirical studies in order to make a more comprehensive assessment of how each study performed for this methodological quality item.

### Statistical Analyses

Eight studies investigated discrepancies in statistical analyses [6],[15],[17],[20],[23],[25],[27],[28]. Table 5 summarises the results of these studies. Three studies reported discrepancies in the analysis methods between documents: discrepancy rates ranged from 7% (3/42) of their included studies to 88% (7/8) [15],[17],[27]. Five studies considered whether an intention-to-treat analysis or per protocol analysis was specified or reported [6],[20],[23],[25],[28]. Three of these studies found discrepancies between different documents (due to the absence of information) in 17% (8/48) to 96% (49/51) of included RCTs [6],[23],[25], and another study [28] found discrepancies (due to a contradiction) between protocols and publications in 67% (2/3) of RCTs. Melander et al. found that only one analysis was reported in 92% (23/25) of RCTs, usually favouring the per protocol analysis [20]. One study [15] found that an equivalence RCT was reported as a superiority RCT, and that there were discrepancies (information absent) in interim analyses in 62% (8/13) of RCTs. Rosenthal and Dwan found discrepancies (contradictions and information absent) between trial registry entries and publications in the reporting of outcomes at different time points [6].

**Table 5:** Statistical analyses.

Study (Maximum Number of RCTs)	Quality^a^	Results	Percent of RCTs (Number)
**Chan, 2008 [15]** ** (** ***n = *** **70)**	H–H	**Trial design:** discrepancy (contradiction) between protocol and publication (equivalence trial reported as a superiority trial)	1% (1/70)
		**Statistical test used for primary outcome:** discrepancy (contradiction) between protocol and publication (statistical methods for analysing the primary outcomes were described in 39 protocols)	64% (25/39)
		**Statistical test used for primary outcome:** discrepancy (information absent) between protocol and publication (statistical methods for analysing the primary outcomes were described in 42 publications)	7% (3/42)
		**Interim analyses:** discrepancy (information absent) between protocol and publication (reported in protocol but not in publication) (interim analyses were described in 13 protocols)	62% (8/13)
		**Interim analyses:** discrepancy (contradiction) between publication and protocol (reported in publication but it was explicitly stated in the protocol that there would be none) (interim analyses were reported in seven publications)	29% (2/7)
**Hahn, 2002 [17]** ** (** ***n = *** **15)**	X–H	**Analysis plan:** discrepancy (contradiction) between protocol and publication (statistical analysis plan was described in only eight protocols)	88% (7/8)
**Turner, 2008 [27]** ** (** ***n = *** **74)**	HHH	**Methods:** discrepancy (contradiction) between FDA review and publication (only 51 of the 74 FDA-registered studies were published)	22% (11/51)
		**Statistical tests:** discrepancy (contradiction) between FDA review and publication	16% (4/51)
**Melander, 2003 [20]** ** (** ***n = *** **42)**	?HH	**ITT/PP analysis:** one analysis included in publication (usually favouring PP) (only 25 of the 42 studies were submitted studies with stand-alone publications)	92% (23/25)
**Vedula, 2013 [28]** ** (** ***n = *** **11)**	HHH	**Definition of ITT:** discrepancy (contradiction) between the protocol and publication (ITT definition described in both protocol and publication for three trials)	67% (2/3)
**Rising, 2008 [23]** ** (** ***n = *** **164)**	HHH	**ITT analysis:** discrepancy (information absent) between NDA and publication (reported in NDA but not in publication)	19% (24/126)
		**ITT analysis:** discrepancy (information absent) between NDA and publication (reported in publication but not in NDA) (only 126 of the 164 NDA trials were published)	18% (23/126)
**Soares, 2004 [25]** ** (** ***n = *** **59)**	?–H	**ITT analysis:** discrepancy between protocol and publication (after verification by the Radiation Therapy Oncology Group) (58 published articles were found for 56 protocols used in this study, and 48 undertook an ITT analysis)	17% (8/48)
**Rosenthal, 2013 [6]** ** (** ***n = *** **51)**	X–H	**ITT/PP analysis:** discrepancy (information absent) between registry and publication (reported in publication but not in registry)	96% (49/51)
		**Time point outcome was measured:** discrepancy between trial registration and publication	10% (5/51)
		**Time point outcome was measured:** Not pre-specified in registry but included in publication (information absent)	14% (7/51)

a Based on summary assessments for three domains from Table 4: H = high quality; X = methodological concerns; ? = uncertain quality; – = not applicable.

FDA, US Food and Drug Administration; ITT, intention to treat; NDA, new drug application; PP, per protocol.

### Composite Outcomes

One study (Table 6) investigated discrepancies in composite outcomes [16] and found changes in the specification of the composite outcome between abstracts, methods, and results sections in 33% (13/40) of RCTs. In 69% (11/16) of RCTs with a statistically significant composite outcome, the abstract's conclusion falsely implied that the effect was also seen for the most important component. This reporting strategy of highlighting that the experimental treatment is beneficial despite a statistically non-significant difference for the primary outcome is one form of “spin” [32].

**Table 6:** Composite outcomes.

Study (Maximum Number of RCTs)	Quality^a^	Results	Percent of RCTs (Number)
**Cordoba, 2010 [16]** ** (** ***n = *** **40)**	H–H	**Composite outcomes:** estimates for both the composite and its components reported in the publication	60% (24/40)
		**Composite outcomes:** discrepancy (contradiction) in specification between the abstract/methods and results sections of the publication	33% (13/40)
		**Composite outcomes:** pre-specified composite was not statistically significant, but the new, post hoc composite was statistically significant (contradiction) (four studies constructed a post hoc composite outcome)	75% (3/4)
		**Composite outcomes:** in trials with a statistically significant composite, the abstract conclusion falsely implied that the effect applied also to the most important component (16 trials had a statistically significant composite outcome)	69% (11/16)

a Based on summary assessments for three domains from Table 4: H = high quality; – = not applicable.

### Handling Missing Data

Three studies investigated discrepancies in the handling of missing data [15],[23],[27] (Table 7). One study found that methods of handling missing data differed between documents in 12% (6/51) of RCTs [27]. Chan et al. found discrepancies in methods between protocols and publications in 80% (39/49) of RCTs, and also that in 78% (38/49) of RCTs that reported methods in the publication, these methods were not pre-specified [15]. Rising et al. [23] found that some studies reported the method in the new drug application but not in the trial publication and vice versa.

**Table 7:** Handling missing data.

Study (Maximum Number of RCTs)	Quality^a^	Results	Percent of RCTs (Number)
**Chan, 2008 [15]** ** (** ***n = *** **70)**	H–H	**Methods of handling missing data:** discrepancy between protocol and publication	80% (39/49)
		**Methods of handling missing data:** discrepancy (information absent) between publication and protocol (reported in publication but not in protocol) (the method of handling missing data was described in 16 protocols and 49 publications)	78% (38/49)
**Rising, 2008 [23]** ** (** ***n = *** **164)**	HHH	**Method of imputation:** discrepancy (information absent) between NDA and publication (LOCF reported in NDA but not in publication)	8% (10/126)
		**Method of imputation:** discrepancy (information absent) between NDA and publication (LOCF reported in publication but not in NDA)	9% (11/126)
		**Method of imputation:** discrepancy (information absent) between NDA and publication (imputation method reported in NDA but not in publication)	12% (15/126)
		**Method of imputation:** discrepancy (information absent) between NDA and publication (imputation method reported in publication but not in NDA) (only 126 of the 164 NDA trials were published)	13% (16/126)
**Turner, 2008 [27]** ** (** ***n = *** **74)**	HHH	**Methods of handling dropout:** discrepancy (contradiction) between FDA review and publication (only 51 of the 74 FDA-registered studies were published)	12% (6/51)

a Based on summary assessments for three domains from Table 4: H = high quality; – = not applicable.

FDA, US Food and Drug Administration; LOCF, last observation carried forward; NDA, new drug application.

### Unadjusted versus Adjusted Analyses

Three studies [15],[24],[29] found discrepancies in unadjusted versus adjusted analyses in 46% (36/79) to 82% (23/28) of RCTs (Table 8).

**Table 8:** Unadjusted versus adjusted analyses.

Study (Maximum Number of RCTs)	Quality^a^	Results	Percent of RCTs (Number)
**Chan, 2008 [15]** ** (** ***n = *** **70)**	H–H	**Adjusted analyses:** discrepancy between protocol and publication (28 trials described adjusted analyses in the protocol or publication)	82% (23/28)
		**Adjusted analyses:** discrepancy (information absent) between protocol and publication (specified in protocol but the publication reported no adjustment or omitted a pre-specified covariate) (18 trials described adjusted analyses in the protocol)	67% (12/18)
		**Covariates:** discrepancy (contradiction) between publication and protocol (published adjusted analyses in publication using covariates that were not pre-specified in the protocol or added a covariate) (18 trials described adjusted analyses in the publication)	67% (12/18)
**Yu, 2010 [29]** ** (for 2000: ** ***n = *** **79; for 2006: ** ***n = *** **109)**	XHH	**Adjusted analyses (2000):** discrepancy between methods and results sections of the publication	46% (36/79)
		**Adjusted analyses (2000):** discrepancy (contradiction) between methods and results sections of the publication (specified adjusted analysis in the methods but reported only unadjusted results in the results)	6% (2/36)
		**Adjusted analyses (2000):** did not specify clearly the type of analysis used in the results section	30% (24/79)
		**Adjusted analyses (2006):** did not specify clearly the type of analysis used in the results section	17% (19/109)
		**Adjusted analyses (2006):** discrepancy (contradiction) between methods and results sections of the publication (specified adjusted analysis in the methods but reported only unadjusted results in the results)	16% (3/19)
**Saquib, 2013 [24]** ** (** ***n = *** **199)**	HHH	**Adjusted analyses:** discrepancy (contradiction) between trial registry/protocol/design paper and publication (comparisons were made for 60 published trials for which information on adjustment was available from the trial registry, protocol, or design paper)	47% (28/60)
		**Adjusted analyses:** discrepancy (information absent) between protocol and publication (adjusted analyses not pre-specified in the protocol but included in the publication)	75% (21/28)
		**Adjusted analyses:** discrepancy (information absent) between publication and protocol (adjusted analyses pre-specified in the protocol but not included in the publication)	25% (7/28)

a Based on summary assessments for three domains from Table 4: H = high quality; X = methodological concerns; – = not applicable.

### Continuous/Binary Data

Two studies investigated discrepancies in the use of continuous and binary versions of the same underlying data [6],[27] (Table 9). Turner et al. [27] found a continuous measure rendered binary in 1% (1/74) of RCTs, and Rosenthal and Dwan [6] found discrepancies between trial registry entries and publications in 29% (12/42) of RCTs. A third study, Riveros et al. [31], found that in 20% (9/45) of RCTs there were discrepancies due to different types of analysis (i.e., change from baseline versus final-value mean) between results posted in trial registry entries at ClinicalTrials.gov and in the corresponding publications. Rosenthal et al. also found discrepancies (contradictions and information absent) for final values versus change from baseline [6].

**Table 9:** Continuous/binary data.

Study (Maximum Number of RCTs)	Quality^a^	Results	Percent of RCTs (Number)
**Turner, 2008 [27]** ** (** ***n = *** **74)**	HHH	**Measurement scale:** continuous measure rendered binary	1% (1/74)
**Rosenthal, 2013 [6]** ** (** ***n = *** **51)**	X–H	**Cutoffs in binary/categorical variables:** discrepancy between trial registry and publication (12 were not included in trial registry but included in publication, and only 42 trials considered cutoffs in binary/categorical variables)	29% (12/42)
		**Final values versus change from baseline:** discrepancy between trial registry and publication (32 trials included continuous outcomes measured at baseline and end point)	3% (1/32)
		**Analysis end point:** Not pre-specified in registry but included in publication (information absent)	41% (13/32)
**Riveros, 2013 [31]** ** (** ***n = *** **202)**	H–H	**Final values versus change from baseline:** discrepancy between trial registry and publication (107 trials included continuous outcomes, and comparisons could not be made for 45 trials)	20% (9/45)

a Based on summary assessments for three domains from Table 4: H = high quality; X = methodological concerns; – = not applicable.

### Subgroup Analyses

Twelve studies investigated discrepancies in subgroup analyses [6],[11]–[15],[18],[19],[21],[22],[26],[30] (Table 10). The majority considered whether subgroup analyses were pre-specified or post hoc. Assmann et al. found that it was commonly difficult to distinguish between these different timings for the choice of subgroup analyses [12]. Four studies considered discrepancies; and the discrepancy rate ranged from 61% (11/18) to 100% (25/25) of RCTs in three studies [11],[14],[15]. The fourth study found subgroup analyses reported in only seven RCTs, and no details had been included in the trial registry entries for six of these RCTs, while the seventh had no discrepancies [6]. In seven studies [11],[13],[18],[21],[22],[26],[30], where the comparison was mostly made between the methods and results sections of the trial publication, it was found that a number of subgroup analyses conducted were not pre-specified (range: 14% [8/58] to 91% [49/54] of RCTs), pre-specified, but not reported in the publication (range: 27% [3/11] to 53% [9/17]), or contained a mixture of pre-specified and non-pre-specified subgroup analyses (range: 10% [6/58] to 65% [135/207]).

**Table 10:** Subgroup analyses.

Study (Maximum Number of RCTs)	Quality^a^	Results	Percent of RCTs (Number)
**Chan, 2008 [15]** ** (** ***n = *** **70)**	H–H	Discrepancy between protocol and publication (25 trials described subgroup analyses in the protocol or publication)	100% (25/25)
		Discrepancy between protocol and publication (pre-specified in protocol and only some or none reported in publication) (13 trials described subgroup analyses in the protocol)	92% (12/13)
		Discrepancy (information absent) between protocol and publication (included in the publication, but at least one not pre-specified in the protocol) (20 trials described subgroup analyses in the publication)	95% (19/20)
**Al-Marzouki, 2008 [11]** ** (** ***n = *** **37)**	?–H	Publication includes at least one unreported or new subgroup analysis not mentioned in the protocol (18 trials pre-specified subgroup analyses in the protocol, 28 trials included subgroup analyses in the publication)	61% (11/18)
		Reason for subgroup selection reported in the protocol	3% (1/37)
		Subgroup analyses reported in the publication but not pre-specified in the protocol (information absent)	58% (11/19)
		Reason for these subgroup analyses reported in the publication	0% (0/11)
**Sun, 2011 [26]** ** (** ***n = *** **207)**	HHH	Not pre-specified (information absent) (64 trials claimed a subgroup effect for the primary outcome)	59% (38/64)
		At least one subgroup analysis not pre-specified	65% (135/207)
**Wang, 2007 [30]** ** (** ***n = *** **59)**	?HH	Number of subgroup analyses undertaken was unclear	15% (9/59)
		Unclear whether any of the subgroup analyses were pre-specified or post hoc	68% (40/59)
		Unclear whether some subgroup analyses were pre-specified	5% (3/59)
**Hernandez, 2006 [18]** ** (** ***n = *** **63)**	HHX	Included pre-specified and non-pre-specified subgroups in the publication	10% (4/39)
		Subgroup analyses reported in the publication without a rationale (39 trials included subgroup analyses in the publication)	54% (21/39)
**Bhandari, 2006 [13]** ** (** ***n = *** **72)**	H–H	Subgroup analyses not pre-specified (information absent) (54 subgroup analyses in 27 trials)	91% (49/54)
**Parker, 2000 [22]** ** (** ***n = *** **67)**	?HH	Subgroup analyses post hoc	14% (8/58)
		partially pre-specified and partially post hoc	10% (6/58)
		Subgroup analyses failed to indicate whether they were pre-specified or not (58 trials reported subgroup analyses)	35% (20/58)
**Hernandez, 2005 ** [19] ** (** ***n = *** **18)**	XXH	Pre-specified subgroups not reported in the publication (information absent)	27% (3/11)
		Partially pre-specified subgroups were reported in the publication (11 trials reported subgroup analyses)	46% (5/11)
		Discrepancy (information absent) between protocol and publication (subgroup analyses not pre-specified in the protocol but reported in publication)	50% (3/6)
		Discrepancy between publication and protocol (subgroup analyses pre-specified in the protocol but not reported in publication as planned) (protocols were available for six trials)	33% (2/6)
**Moreira, 2001 [21]** ** (** ***n = *** **32)**	X–H	Subgroup analyses unclear	6% (1/17)
		Subgroup analyses omitted from the publication (information absent) (17 trials had at least one subgroup analysis)	53% (9/17)
		Subgroups defined after randomisation (14 trials reported when subgroups were defined in the publication)	82% (4/14)
**Boonacker, 2011 [14]** ** (** ***n = *** **79)**	?HH	Discrepancy between the grant proposal and publication (47 were RCTs only)	77% (36/47)
		Discrepancy between the grant proposal and publication	75% (59/79)
		Discrepancy in pre-specified subgroup analyses between the grant proposal and publication	90% (44/49)
		Discrepancy (information absent) in pre-specified subgroup analyses between the grant proposal and publication (pre-specified subgroups not reported)	22% (11/49)
		Discrepancy in pre-specified subgroup analyses between the grant proposal and publication (added/omitted subgroups from publications) (49 studies mentioned subgroups in their grant application)	67% (33/49)
		Subgroups included in the publication (30 did not pre-specify subgroups)	50% (15/30)
		No discrepancy between protocol and grant proposal	62% (13/21)
		No discrepancy between protocol and publication (a protocol was available for 21 trials)	38% (8/21)
		Subgroup effects were reported only for significant interaction tests (four publications included interaction tests)	75% (3/4)
**Rosenthal, 2013 [6]** ** (** ***n = *** **51)**	X–H	Discrepancy between trial registry and publication	0% (0/7)
		Subgroup analyses not included in trial registry but included in publication (information absent) (seven trials included subgroup analyses in trial registry or publication)	86% (6/7)
**Assmann, 2000 [12]** ** (** ***n = *** **50)**	H–H	It was commonly difficult to determine whether subgroup analyses were pre-defined or post hoc	Not applicable

a Based on summary assessments for three domains from Table 4: H = high quality; X = methodological concerns; ? = uncertain quality; – = not applicable.

### Funding

Although 11 studies looked at funding as a study characteristic, only two considered the relationship between discrepancies and funding. Sun et al. found that trials funded by industry were more likely to report subgroup results when the primary outcome was not statistically significant compared to when the primary outcome was statistically significant (odds ratio 3.00 [95% CI: 1.56 to 5.76], *p* = 0.001) [26].

Rosenthal and Dwan found no statistically significant differences in discrepancy rates of primary and secondary outcomes between registry entries and final reports of industry-sponsored versus non–industry-sponsored trials [6].

## Discussion

### Summary of Main Results

Twenty-two cohort studies of RCTs were included in this review that examined discrepancies between information given in different sources. Many different types of discrepancies in analyses between documents were found, and discrepancy rates ranged from 7% (3/42) to 88% (7/8) for analysis methods, 46% (36/79) to 82% (23/28) for the presentation of adjusted versus unadjusted analyses, and 61% (11/18) to 100% (25/25) for subgroup analyses.

None of the included studies examined the selective reporting bias of analyses in RCTs that would arise if analyses were reported or concealed because of the results. Such an assessment may prove to be difficult without access to statistical analysis plans and trial datasets to determine the statistical significance of the results for the analyses that were planned and for those that were reported.

The majority of studies (12) focussed on the reporting of subgroup analyses. None of the included studies provided any detail on the reasons for inconsistencies. A number of studies commented on whether or not reported subgroup analyses were pre-specified. While this may not be seen as a comparison, we reported the findings for these studies within this review because post hoc decisions about which subgroups to analyse and report may be influenced by the findings of those or related analyses. There are likely to be many other selective inclusion and reporting mechanisms for which there is no current empirical evidence, and a more complete categorisation is provided elsewhere [4]. The methodological concerns that were observed in the included studies were not critical, and they should not impact importantly on the interpretation of the results of the studies.

These discrepancies may be due to reporting bias, errors, or legitimate departures from a pre-specified protocol. Reliance on the source documentation to distinguish between these reasons may be inadequate, and contact with trial authors may be necessary. Only one study [29] contacted the original authors of the RCTs for information about the discrepancies, but only 9% (3/34) of those authors replied, and no details were given on the reasons for the discrepancies. In terms of selective reporting of outcomes, a previous study interviewed a cohort of trialists about outcomes that were specified in trial protocols but not fully reported in subsequent publications [33]. In nearly a quarter of trials (24%, 4/17) in which pre-specified outcomes had been measured but not analysed, the “direction” of the main findings influenced the investigators' decision not to analyse the remaining data collected.

### Agreements and Disagreements with Other Studies or Reviews

Three of the included studies [11],[17],[25] were included in a previous Cochrane methodology review that was restricted to studies that compared any aspect of trial protocols or trial registry entries to publications [5]. The conclusions of the current review and the previous review are similar in that discrepancies were common, and reasons for them were rarely reported in the original RCTs. This current review focussed on analyses only, and included studies that compared different pieces of trial documentation or details within a trial publication.

### Implications for RCTs

In accordance to International Conference on Harmonisation of Technical Requirements for Registration of Pharmaceuticals for Human Use E 9 guidance (*Statistical Principles for Clinical Trials* [34]), procedures for executing the statistical analysis of the primary, secondary, and other data should be finalised before breaking the blind on those analyses. The availability of a trial protocol (or separate statistical analysis plan) is of prime importance for inferring whether the results presented are a selected subset of the analyses that were actually done and whether there are legitimate reasons for departures from a pre-specified protocol. Many leading medical journals, e.g., *PLOS Medicine* (http://www.plosmedicine.org/static/guidelines.action) and the *British Medical Journal* (http://www.bmj.com/about-bmj/resources-authors/article-submission/article-requirements), now require the submission of a trial protocol alongside the report of the RCT for comparison during the peer review process. In order to ensure transparency, protocols and any separate analysis plans for all trials need to be made publicly available, along with the date that the statistical analysis plan was finalised, details of reasons for any changes, and the dates of those changes. Additional analyses suggested by peer reviewers when a manuscript is submitted for publication should be judged on their own merits, and any additional analyses that are included in the final paper should be labelled as such.

Whilst evidence-based guidelines exist for researchers to develop high-quality protocols for clinical trials (e.g., SPIRIT [35]) and for reporting trial findings (CONSORT [36]), more guidance is needed for writing statistical analysis plans.

### Implications for Systematic Reviews and Evaluations of Healthcare

Systematic reviewers need to ensure they access all possible trial documentation, whether it is publicly available or obtained from the trialists, in order to assess the potential for selective reporting bias for analyses. The Cochrane risk of bias tool is currently being updated, and the revised version will acknowledge the possibility of selective analysis reporting in addition to selective outcome reporting. Selective analysis reporting generally leads to a reported result that may be biased, so sits more naturally alongside other aspects of bias assessment of trials, such as randomisation methods, use of blinding, and patient exclusions. Selective outcome reporting may lead either to bias in a reported result (e.g., if a particular measurement scale is selected from among several) or to non-availability of any data for a particular outcome (e.g., if no measures for an outcome are reported). The latter sits more naturally alongside consideration of “publication bias” (suppression of all information about a trial).

### Conclusions

There are to date no readily accessible data on selective reporting bias of analyses in cohorts of RCTs. Studies that have compared source documentation from before the start of a trial with the final trial publication have found a number of discrepancies in the way that analyses were planned and conducted. From the published literature, there is insufficient information to distinguish between bias, error, and legitimate changes. Reliance on the source documentation to distinguish between these may be inadequate, and contact with trial authors may be necessary. If journals insisted that authors provide protocols and analysis plans, selective reporting would be more easily detectable, and possibly reduced. Journals could flag research articles that provide no protocol or statistical analysis plans. More guidance is needed on how a detailed statistical analysis plan should be written.

## Supporting Information

Text S1 **PRISMA statement.**
(DOC)

Text S2 **Search strategies.**
(DOCX)

## Abbreviations

RCT: randomised controlled trial
