# Supplementary material for: Evidence for the Selective Reporting of Analyses and Discrepancies in Clinical Trials: A Systematic Review of Cohort Studies of Clinical Trials
Source: PLoS Med. 2014 Jun 24;11(6):e1001666. doi: 10.1371/journal.pmed.1001666 (PMC4068996; doi:10.1371/journal.pmed.1001666)
Supplement: Text S2 — Search strategies. (DOCX) [file pmed.1001666.s002.docx]

**Search strategies**

**Search of Cochrane Methodology Register (Wiley) (1878 to 28^th^ May 2013 (last update of database was July 2012)):**

1. (reporting bias):kw or (selective reporting):kw in Methods Studies
2. selective* report* in Methods Studies
3. selective reporting bias in Methods Studies
4. #1 or #2 or #3

**Search of PubMed (1950 to 5^th^ February 2014):**

1. selective* report* [tiab]
2. selective non reporting [tiab]
3. selective non-reporting [tiab]
4. biased report [tiab]
5. biased reporting [tiab]
6. biased publishing [tiab]
7. biased publication [tiab]
8. selective reporting bias [tiab]
9. Randomized Controlled Trial as Topic [MeSH: noexp]
10. Clinical Trials as Topic [MeSH: noexp]
11. (randomised OR randomized OR rct OR rcts OR trial*) [tiab]
12. #1 or #2 or #3 or #4 or #5 or #6 or #7 or #8
13. #9 or #10 or #11
14. #12 and #13

**Search of PsycInfo (Ovid) (1887 to 5^th^ February 2014):**

1. AB selective reporting bias
2. KW selectiv* report*
3. KW selective reporting bias
4. AB (randomised OR randomized OR rct OR rcts OR trial*)
5. #1 OR #2 OR #3
6. #4 AND #5

**Search of Medline (Ovid) (1948 to 5^th^ February 2014):**

1. selective reporting bias.mp.
2. selectiv$ report$.mp.
3. (randomised or randomized or rct or rcts or trial$).mp.
4. 1 or 2
5. 3 and 4
